# In-House Validation of an Efficient and Rapid Procedure for the Simultaneous Determination and Monitoring of 23 Mycotoxins in Grains in Korea

**DOI:** 10.3390/toxins14070457

**Published:** 2022-07-02

**Authors:** Hyoyoung Kim, Eun Joo Baek, Byeung Gon Shin, Ho Jin Kim, Jang-Eok Kim

**Affiliations:** 1 Experiment Research Institute, National Agricultural Products Quality Management Service, 141, Yongjeon-ro, Gimcheon-si 39660, Korea; hyo02@korea.kr (H.K.); qrgh1004@gmail.com (E.J.B.); sbkon1@korea.kr (B.G.S.); 2 Major in Environment and Life Chemistry, School of Applied Biosciences, College of Agriculture and Life Sciences, Kyungpook National University, Daegu 41566, Korea

**Keywords:** mycotoxins, monitoring, LC-MS/MS, multi-mycotoxin method, grains, ergot alkaloids

## Abstract

A high-performance liquid chromatography tandem mass spectrometry method is described for the simultaneous determination of mycotoxins, including Ergot alkaloids (EAs), in 3 types of grains. The extraction of 23 mycotoxins was evaluated and performed by using a modified QuEChERS-based sample preparation procedure. The proposed method was fully validated on spiked grain samples (barley, wheat and oat) to assess the linearity, limit of detection (LOD) and limit of quantitation (LOQ), matrix effects, precision and recovery. After validation, this method was applied to 143 samples of various types of 3 grains from the Republic of Korea to survey the level of mycotoxin contamination in Republic of Korean grains. A total of 42 grain samples (29%) were contaminated with at least one of these mycotoxins at levels higher than the LOQ. The results demonstrated that the procedure was suitable for simultaneously determining these mycotoxins in cereals and could be performed for their routine analysis in mycotoxin laboratories.

## 1. Introduction

Mycotoxins are secondary metabolites produced by filamentous fungi, such as those belonging to the genera *Claviceps*, *Aspergillus*, *Fusarium* and *Penicillium*, and have been ranked as the most important chronic dietary risk factor, higher than synthetic contaminants, plant toxins, food additives or pesticide residues [1,2,3,4,5]. Fungal toxins pose harmful effects to human and animal health, such as hepatotoxicity, nephrotoxicity, reproductive problems, immunosuppression and carcinogenicity [6,7,8,9]. Grains are the most susceptible crops to fungal contamination and mycotoxins, the main mycotoxins being aflatoxins (AFLs), ochratoxin A (OTA), deoxynivalenol (DON), T-2 toxin (T-2), HT-2 toxin (HT-2), fumonisin (FB), zearalenone (ZEN) and ergot alkaloids (EAs) [10,11,12]. According to the annual report of the Rapid Alert System for Food and Feed (RASFF), grains are the most affected by fungal contamination, and the problem with mycotoxins is the main problem with the most reported type of risk [13].

Maximum limits have been established for these mycotoxins in grain to reduce risks for consumers. Briefly, in the European Commission (EC) 1881/2006 [14], all grains except maize were set to no more than the sum of AFLs (B_1_, B_2_, G_1_ and G_2_) 4 μg/kg. The DON was 1750 μg/kg in unprocessed durum wheat and oats. For unprocessed grains, OTA and ZEN (except maize) were set at 5 μg/kg and 100 μg/kg, respectively. The sum of HT-2 and T-2 toxins should be less than 200 μg/kg in malted barley according to EC 2013/165/EU [15]. Following EC 1881/2006 in revised 2022, the maximum levels for ergot alkaloids were proposed [14]. The maximum levels will apply to the sum of the following 12 alkaloids: ergometrine, ergosine, ergocornine, ergotamine, ergocristine, ergocryptine (α- and β-form) and their respective -inine forms. For barley, wheat, spelt and oat milled products with an ash content of less than 900 mg/100 g, the limit is not more than 100 μg/kg, and for milled products with an ash content of 900 mg/100 g or more, the limit is not more than 150 μg/kg.

In fact, a number of methods exist for the analysis of mycotoxins. Most methods are based on high-performance liquid chromatography-ultraviolet light (HPLC-UV) [16,17], high-performance liquid chromatography-fluorescence detection (HPLC-FLD) [18,19,20], and liquid chromatography-evaporative light scattering detector (LC-ELSD) [21,22]. However, these methods have several drawbacks, particularly at low residual levels, causing false-positives [23]. Therefore, HPLC combined with tandem mass spectrometry (MS/MS) is a highly sensitive, specific and reliable tool for detecting contaminants in food, and has become the most popular approach for multianalyte analyses [10,24].

Extraction and purification are performed before injection, and purification by IAC after extraction is common [25,26]. The immunoaffinity column (IAC) method, which mainly uses specific antibodies to mycotoxins, was used [27]. Commercial IACs have several disadvantages, such as a low mycotoxin recovery, variable and expensive costs, a large amount of time required, the use of toxic solvents, such as chlorinated solvents, and sometimes requiring practical experience [28].

The QuEChERS (acronym of Quick, Easy, Cheap, Effective, Rugged, and Safe) procedure is fast and easy to perform, requires minimal amounts of chemicals (especially solvents), covers a wide range of analyte-matrix pairs, and provides some degree of selectivity [29]. Therefore, we modified and extracted QuEChERS and then used MS/MS to validate the mycotoxin in three matrices.

To the best our knowledge there is no study that conducted a simultaneous analysis and validation of 23 mycotoxins, including ergot alkaloids, and it can be assumed that grain contamination by mycotoxins is similar worldwide. However, there are still no results of mycotoxin monitoring on grains grown in the Republic of Korea. Therefore, the objective of the present study was to develop a fast and sensitive method for the simultaneous quantification of 23 mycotoxins, including ergot alkaloids, using HPLC coupled to an MS/MS detector after QuEChERS-based extraction in 3 matrices (barley, wheat, oat) to avoid additional cleanup steps.

The proposed method was fully validated on spiked grain samples (barley, wheat and oat) to assess the linearity, limit of detection (LOD) and limit of quantification (LOQ), matrix effects, precision and recovery. After validation, this method was applied to 143 samples of various types of 3 grains (barley, wheat, oat) from the Republic of Korea to survey the degree of mycotoxin contamination, including EAs, in Republic of Korean grains.

## 2. Results and Discussion

### 2.1. Optimization of the HPLC-MS/MS Method

**Figure 1:**
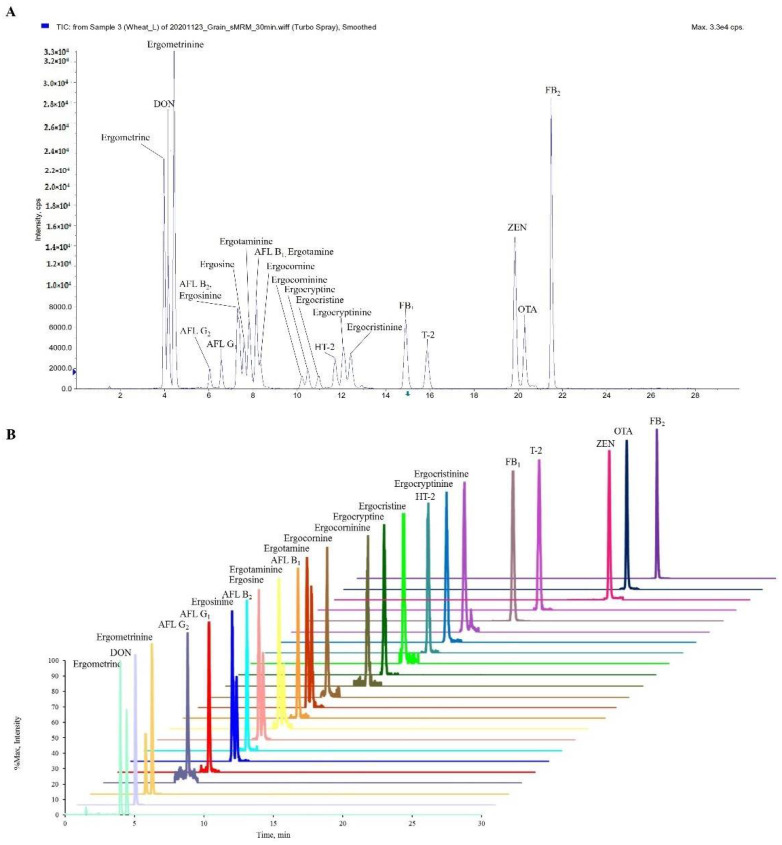
(**A**) Total ion chromatograms and (**B**) extracted ion chromatograms of the 23 mycotoxins added to blank wheat samples (2 μg/kg for AFL B_1_, AFL B_2_, AFL G_1_, AFL G_2_; 80 μg/kg for FB_1_, FB_2_; 5 μg/kg for OTA; 200 μg/kg for DON; 20 μg/kg for ZEN; 10 μg/kg for HT-2, T-2; 4 μg/kg for EAs).

The chemical structures of 23 types of mycotoxins are shown in Figure S1. Initially, a full scan and MS/MS spectra of all mycotoxins were obtained by injecting individual standard solutions diluted with an ACN:water (50:50, *v*/*v*) solution into the mass spectrometer. All the mycotoxins were tested using both the ESI+ and ESI− modes. The parent ion and product ions, retention time, declustering potential (DP), collision energy (CE), collision cell entrance potential (CEP) and collision cell exit potential (CXP) of all the compounds are shown in Table S1. ESI+ showed the best results in terms of the sensitivity for most of the mycotoxins, and protonated precursor ions [M + H]+ were selected for most of them, whereas ZEN was evaluated in ESI− mode and was detected in the deprotonated [M − H]− form. Each compound was characterized by its retention time and its two product ions. On the basis of the guidelines outlined in Document N°SANTE/11312/2021 [30] the most intense product ion was used as a quantitative ion, while the second product ion with its specific retention time was used for confirmation. The two characteristic product ions in extraction ion chromatography must overlap completely, and the responses of the two ions have a sufficient sensitivity and selectivity in the linear range. For the precursor ion, 6 ergot alkaloids (ergometrine, ergosine, ergotamine, ergocornine, ergocryptine, ergocristine) and each isomer have the same proton ion. For example, ergotamine and ergotaminine are isomers, which have the same protonated ions at *m*/*z* 582.0; ergocornine and ergocorninine are isomers, and have the same protonated ions at *m*/*z* 562.1. Figure 1A shows the total ion chromatograms (TICs) of 23 mycotoxins spiked in a blank wheat sample by HPLC-MS/MS. According to the extracted-ion chromatogram, the separation of all target mycotoxins was completed in 30 min (Figure 1B). After different experiments using ESI+ and ESI− and changing multiple reaction monitoring (MRM) conditions, differentiation between the 23 analytes was achieved via MS/MS detection (Figure S2). 

### 2.2. Evaluation of Extraction and Clean-Up

In this study, three methods for mycotoxin clean-up were compared. First, in the QuEChERS procedure, extraction under acidic conditions using ACN containing modified 5% formic acid [31] was used.

**Figure 2:**
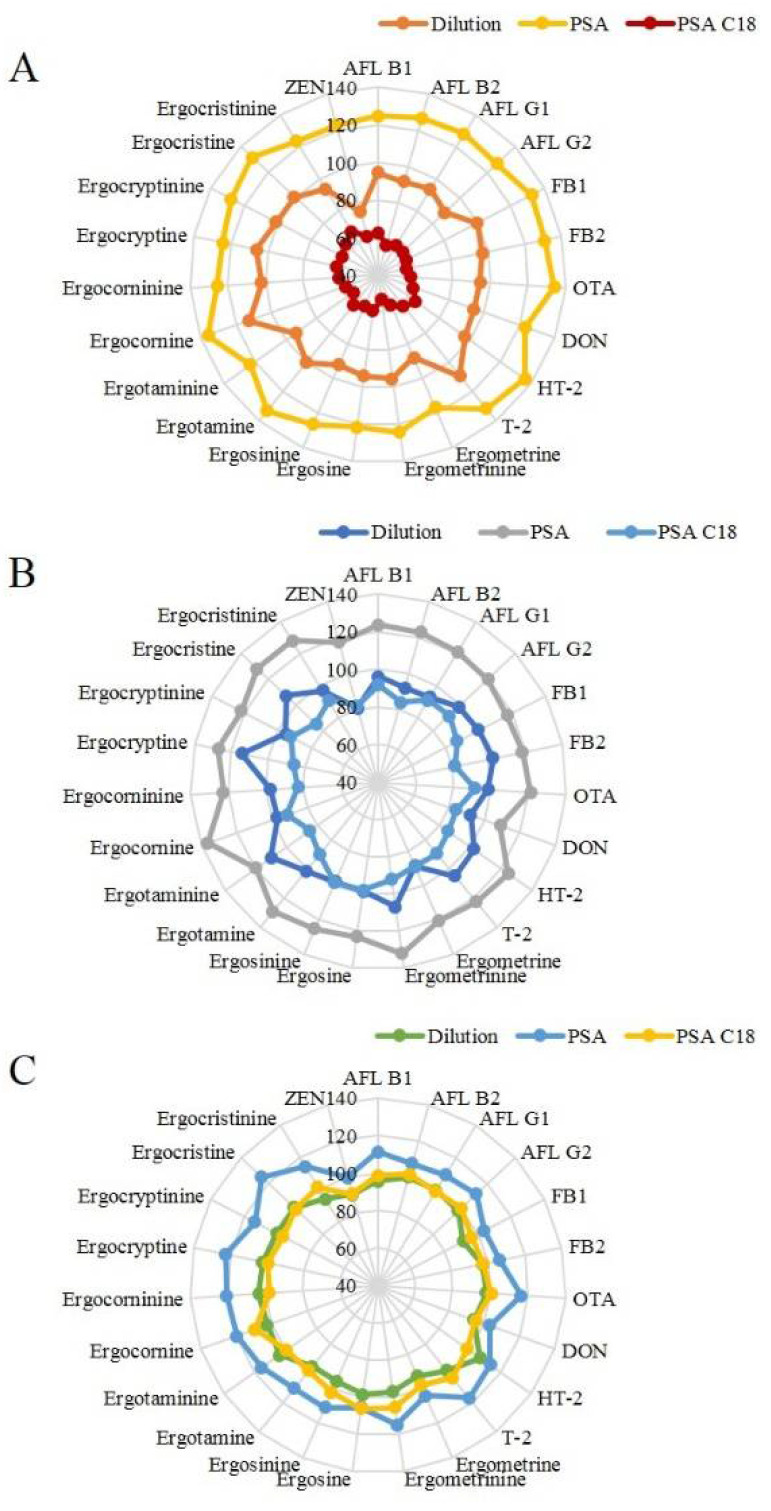
Optimization of the purification method: purification effects with different single filters for (**A**) barley, (**B**) wheat, and (**C**) oat.

Dilutions and purifications (PSA and PSA C18) were selected and compared in order to obtain the best recovery and reduce any matrix effects. As shown in Figure 2A–C, PSA showed a relatively high recovery in all media, and PSA C18 showed the lowest recovery in barley. Satisfactory recovery (74.8 to 114.2%) of all analytes by grains was achieved using dilution, while PSA showed higher recoveries ranging from 99.6 to 136.4% in all matrices. For PSA C18, barley showed the lowest recovery rate of 53–66.5%.

PSA is a weak anion exchanger adsorbent with the ability to remove fatty acids, sugars and other components that form hydrogen bonds, while C18 is used to remove lipids and sterols. Nevertheless, C18 strongly retains planar alkaloids, leading to the low recovery of alkaloids [32,33]. Dilution is often used to lower the matrix effect [4]. As a result, the purification process was omitted to shorten the extraction time and simplify the steps [31]. Sample preparation was quick and simple with the salting-out liquid-liquid extraction (SO-LLE) method, including the dilution process.

### 2.3. Evaluation of the Matrix Effects

Molecules that elute or are coextracted with other impurities during the extraction process alter the ionization efficiency of the analyte, resulting in a different MS response of the analyte in the sample and the interference with the analyte determination [34]. Indeed, the analyte shows a relatively low signal due to interference in the medium compared to in the pure solvent. These phenomena are called matrix effects [23]. 

The grain (barley, wheat, oat) matrix effects were evaluated according to the equations using signals from matrix-matched standards and standards prepared in matrix-free solvents compared under identical conditions. Matrix effects (%) are expressed as a percentage of the difference in the slopes of the corresponding calibration curves in the solvent and matrix. Negative values indicate signal suppression, and positive values indicate signal enhancement [35]. Depending on this percentage value, the matrix effects can be soft (−20% to 0% or 0% to 20%), medium (−51% to −21% or 21% to 50%), and strong (less than −51% or +51%) [36].

**Figure 3:**
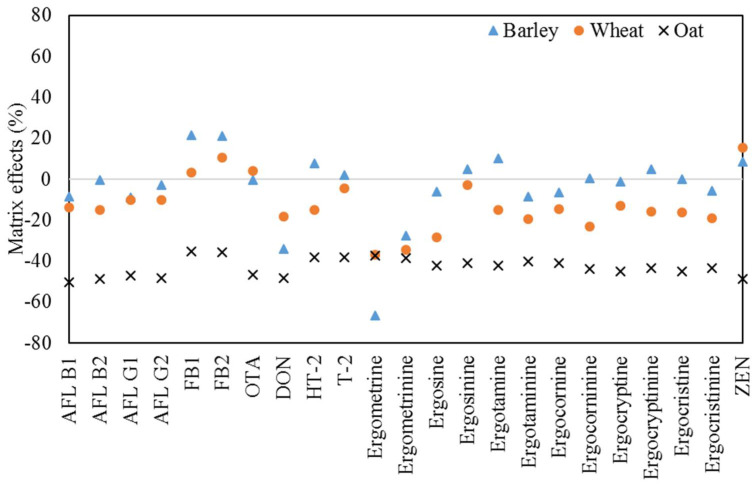
Matrix effects for the 23 analyzed mycotoxins in barley, wheat and oat.

As shown in Figure 3, matrix effects were observed in various matrices, such as barley, wheat, and oat. As can be seen, mycotoxins have large fluctuations in matrix effects depending on the type of grain. In barley, a strong decrease in the slope of the calibration curve was observed for ergometrine (67%), and a medium signal decrease was observed for ergometrinine and DON (28 and 34%, respectively). A medium signal increase in FB was observed from 21 to 22%, but soft matrix effects were observed for other compounds. In the case of wheat, ergometrine, ergometrinine, ergosine, and ergocornine showed a medium signal decrease of 28 to 37%, and most other ingredients showed a soft signal decrease, but only FB, OTA, and ZEN showed a soft signal decrease of 3 to 15%. For oats, all analytes had matrix effects with medium signal reductions (35–50%). Earlier eluted peaks, such as DON, ergometrine, and ergometrinine, showed higher signal suppression, whereas the late eluted peaks showed a less pronounced signal reduction. A previous study showed similar results. In oats and wheat, most of the analytes showed a soft signal decrease, but ergometrine showed a signal decrease of more than 50% in wheat, barley, and oats [37]. In another study, ergometrine showed a significant signal decrease in wheat [10]. Therefore, matrix-matched corrections were used to ensure the accurate quantification of mycotoxins in cereals.

### 2.4. Method Validation

To evaluate the suitability of the proposed method for determining mycotoxins in barley, wheat and oat matrices, the analytical parameters of the HPLC-MS/MS method were validated under conditions according to the guidelines in SANTE/126823/2019 [30].

Table 1 summarizes the calibration curve, linearity, coefficient of determination, LOD, LOQ, recovery and precision results for barley, wheat and oats. Satisfactory coefficients of determination (R^2^ > 0.98) were obtained, confirming that all assay responses were linear across the study range. The slope of mycotoxin according to the medium was lowest in oats, except for ergometrine and ergosine. It showed a low LOQ value for most mycotoxins; that is, the calculated LOQ ranged from 0.1 to 6.34 μg/kg for barley, 0.12 to 5.84 μg/kg for wheat and 0.12 to 7.15 μg/kg for oat. The LOQ levels of mycotoxins in barley, wheat and oats were AFL (0.12 to 0.20 μg/kg), DON (2.23 to 7.15 μg/kg), OTA (0.32 to 0.41 μg/kg), ZEN (0.49 to 1.32 μg/kg), FB (2.89 to 6.34 μg/kg), HT-2 and T-2 (0.31 to 0.83 μg/kg), and EAs (0.10 to 0.39 μg/kg), which were below the maximum levels prescribed for direct human consumption (Table 2) [14]. The intra-day and inter-day RSDs were less than 20%, indicating that this method can be used for the routine analysis of mycotoxins in grains. The concentration interval and recovery rate experimental concentration used for the calibration curve were tested with the concentrations listed in Table S2. Overall, the recovery rates varied from 70.1 to 93.3% in barley, 70.4 to 104.7% in wheat and 70.3 to 109.3% in oats, all with RSD values below 12%. The recovery and RSD met acceptable criteria for all three grain matrices.

### 2.5. Application to Real Samples

A total of 143 samples were analyzed, while a total of 42 samples presented detectable levels of mycotoxins (Table S3). A 34 of the 95 barley samples (36%), 7 of the 19 wheat samples (37%) and 1 of the 29 oat samples (3%) were positive. Among the positive sample types, barley was positive most, with 34 out of 42 (81%). Table 2 shows the range with minimum and maximum values of each positive mycotoxin. In the 95 barley varieties, DON, HT-2 and ZEN predominate, with frequencies of 20 (21%), 15 (16%) and 8 (8%), and mean contamination levels of 27.84, 0.62 and 5.35 μg/kg in barley, respectively. The mycotoxin positive at the highest levels was DON at 112.72 μg/kg in a sample.

**Figure 4:**
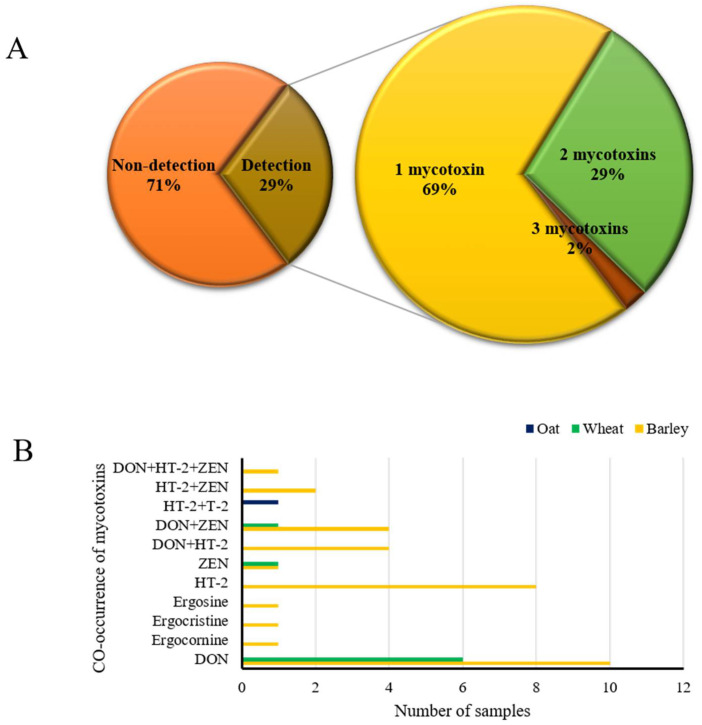
(**A**) Frequency of co-occurrence combinations in positive samples, (**B**) co-occurrence of mycotoxins at levels > LOQ in samples of grains collected in the Republic of Korea.

Mycotoxin occurrence data are presented in Figure 4A of the positive samples, 29% were individually positive for DON, ergocornine, ergocristine, ergosine, HT-2 and ZEN. The most frequent co-contamination occurrence was for 2 (29%), followed by combinations of 3 mycotoxins (2%). Among the 42 detection samples, in the binary combination, DON and ZEN and DON and HT-2 were the most frequently observed combinations at 5 and 4, respectively. A combination of the three mycotoxins was positive in only one sample containing DON, HT-2 and ZEN (Figure 4B). According to these were positive combinations, they belong to mycotoxins mainly produced by the *Fusarium* species. As a result, the levels of all mycotoxins were below the maximum levels (ML) set by the European Commission for barley, wheat and oats [14].

## 3. Conclusions

The HPLC-MS/MS method using ESI+ and ESI- in MRM mode was developed for the fast and simultaneous determination of 23 mycotoxins in grains. The validation data, including linearity, LOD, LOQ, precision and recovery, showed that this method is acceptable for mycotoxin determination in such grains as barley, wheat and oat. The LOD and LOQ ranged from 0.03 to 2.17 μg/kg and 0.1–7.15 μg/kg, respectively. The results also showed that 29% of the grains harvested in the Republic of Korea were contaminated with at least one of the mycotoxins; however, none of the samples exceeded the proposed European regulatory levels. The results indicated that the newly developed and validated HPLC-MS/MS method can be applied to the trace analysis of multicomponent mycotoxin contaminants, including EAs, in grains.

## 4. Materials and Methods

### 4.1. Standard

The standard solutions were prepared as follows: AFB_1_ (1.2 µg/mL), AFG_1_ (1.05 µg/mL), AFB_2_ (1.01 µg/mL) and AFG_2_ (1.04 µg/mL) in acetonitrile (ACN); FB_1_ (50.3 µg/mL) and FB_2_ (50.1 µg/mL) in ACN/water (50/50, *v*/*v*); OTA (10.05 µg/mL) in ACN; ZEN (100.2 µg/mL) in ACN; HT-2 (100.1 µg/mL) in ACN; T-2 (100.0 µg/mL) in ACN; and DON (100 µg/mL) in ACN (Romer Laboratory Diagnostic, Tulln, Austria). Fine film dried ergot alkaloid standards ergometrine, ergosine, ergotamine, ergocornine, ergocryptine, ergocristine and their respective epimers (ergometrinine, ergosinine, ergotaminine, ergocorninine, ergocryptinine, ergocristinine) were purchased from Romer Laboratory Diagnostic (Tulln, Austria). The film-dried standards were reconstituted in 5 mL of ACN to give a concentration of 100 mg/mL for each -ine epimer and 25 mg/mL for each -inine epimer. All stock solutions were stored at −18 °C except for AF and EAs, which were stored at 4 °C.

### 4.2. Chemicals and Reagents

Methanol (MeOH) and acetonitrile were HPLC grade and were both purchased from Merck (Darmstadt, Germany). Ammonium carbonate used in the sample extraction was purchased from Merck (Darmstadt, Germany). HPLC-grade formic acid was purchased from Fisher Chemical (Toronto, ON, Canada). Ammonium formate for LC/MS was obtained from Honeywell (Charlotte, NC, USA). Magnesium sulfate (MgSO_4_) and sodium chloride (NaCl) were used to extract salt, and primary and secondary amine (PSA), C18 and MgSO_4_, which were used for clean-up, were purchased from Bekolut GmbH & Co (Hauptstuhl, Germany). Direct-Q^®^3 UV with pump devices from Millipore (Bedford, MA, USA) was used to purify the demineralized water. A polytetrafluoroethylene (PTFE) syringe filter from Whatman (Maidstone, UK) was used for filtration.

### 4.3. Sample Preparation

#### 4.3.1. Collection of Samples

19 samples of wheat, 29 samples of oat, and 95 samples of barley harvested in the Republic of Korea in 2019 were purchased. The samples were ground in a Blixer 5 (Robot coupe, Vincennes, France) and sieved with a 40 mesh sieve. Immediately after sieving, a portion of approximately 500 g was randomly taken, sealed in a plastic box, and stored at −4 °C. The remaining samples were stored at −20 °C and used for a further analysis.

#### 4.3.2. Extraction of Samples

Sample extraction was based on the first step (extraction/partition process) of the QuEChERS procedure [38]. A 5 g portion of the grain sample and 10 mL of distilled water were placed into a 50 mL polypropylene Falcon tube, which was shaken using a shaker for 1 min. Then, 10 mL of 5% formic acid in ACN was added to the tube and shaken using a shaker for 1 min. A mixture of salts (4 g MgSO_4_ and 1 g NaCl) was added, and the tube was vigorously shaken using a shaker for 1 min. Subsequently, the tube was centrifuged for 5 min at 3500 rpm, and the supernatant was filtered through a 0.22 μm PTFE membrane and transferred to an amber glass vial for HPLC-MS/MS analysis.

### 4.4. HPLC-MS/MS Conditions

A chromatographic analysis was performed on an HPLC Nanospace SI-2 (Shieido, Tokyo, Japan) equipped with a Thermo Scientific™ Syncronis™ aQ C18 column (3 × 100 mm, 3 μm). The column oven temperature was maintained at 40 °C, and the injection volume was 10 μL. Mobile phase A (water with 0.1% formic acid and 0.15 mmol/L ammonium formate) and mobile phase B (MeOH with 0.1% formic acid and 0.15 mmol/L ammonium formate) were used at a flow rate of 0.25 mL/min. A gradient elution was applied as follows: 0.0–1.0 min: 85–50% A; 1.0–15.0 min: 50–40% A; 15.0–15.1 min: 40% A; 15.1–25.0 min: 40–0% A; 25.0-25.1 min: 0–85% A; 25.1-30.0 min: 85% A. The HPLC was coupled to a QTRAP^®^4500 mass spectrometry system (SCIEX, Germany) equipped with an electrospray ionization (ESI) interface. The parameters were as follows: polarity: positive and negative; interface temperature: 450 °C; ion spray voltage: 5500 V; curtain gas: 30 psi; ion source gas 1: 40 psi; ion source gas 2: 60 psi; collision gas (N_2_): 9 psi; and entrance potential (EP): 10 V. After selecting the precursor ions for each analyte, the product ions were obtained with a combination of cone voltage and collision energy (CE), which were previously optimized parameters. The MS characterization molecular ions were selected as precursor ions, and the most abundant transition of the two product ions was used for quantification and the other for confirmation purposes.

### 4.5. Preparation of Mixed Standard Solutions and the Calibration Curve

The concentrations of the mixed standard solutions of 23 mycotoxins were prepared as follows: AF (0.25 mg/L); FB (10 mg/L); OTA (0.625 mg/L); ZEN (2.5 mg/L); HT-2 and T-2 (1.25 mg/L); DON (25 mg/L); and 12 EAs (0.5 mg/L). A standard solution of each mycotoxin was mixed in a 10 mL volumetric flask, and the solution was filled with acetonitrile. The concentrations correspond to concentrations of 1, 40, 2.5, 10, 5, 5, 100 and 2 mg/kg, respectively, in the sample. The concentration of the calibration curve was prepared at the concentration mentioned in Table S2. All solutions were stored in dark brown glass vials in a freezer at −18 °C in the dark.

For the calibration curve, the mixed standard solution was added to the test solution of the extracted sample to minimize the influence of the medium characteristics of each sample. Blank samples were treated as described in the section “Extraction of samples” to obtain a blank matrix solution. Matrix-matched calibration curves were then prepared by combining the mixed standard solution with the blank matrix solution.

### 4.6. Matrix Effects

The matrix effect in the MS/MS detection was investigated by comparing the response of the matrix-assisted standards (blank samples of barley, wheat and oat) and the calibration standards prepared in the solvent (ACN). A prior analysis of the barley, wheat and oat samples used in matrix-matched calibration curves was performed to ensure that they did not contain any of the analytes. Analytical curves were then obtained from five concentrations for each mycotoxin. Matrix effects, such as signal suppression or enhancement, were calculated according to the equation:

$$Matrix\;effects\;\left(\%\right)=\left(100\;*\;\frac{A}{B}\right)-100$$

where *A* is the slope of the analytical curve performed in the matrix (barley, wheat or oat) and *B* is the slope of the curve performed in the solvent.

### 4.7. Method Validation

Validation parameters of linearity, recovery rate, and precision were evaluated according to the guidelines of the SANTE/11312/2021 [30]. Linearity was evaluated through the coefficient of determination (R^2^) of the calibration curves. The recovery rate was determined by adding the mixed standard solution to each blank test sample. The three concentrations of each compound were used to determine the recovery and precision (Table S2). The intra-day (*n* = 3) and inter-day (*n* = 9) of the instrumental method were estimated by determining the relative standard deviations (RSDs, %) via repeated analysis of spike matrix extracts. The LOD and limit of LOQ were calculated as follows through the calibration curve by applying the guidelines of International Conference on Harmonization (ICH) [39].

$$\chi LOD=3.3\;*\;\frac{\sigma }{S}$$

$$\chi LOQ=10\;*\;\frac{\sigma }{S}$$

*σ*: standard deviation of blank (pseudo-blank) signals, *S*: slope of the calibration curve.

### 4.8. Statistical Analysis

All sample measurements were performed in independent triplicate injections. All statistical procedures were performed using MS Excel 2013 (Redmond, WA, USA), and the measurement results were reported as the mean or percentage ± relative standard deviation (RSD). The 23 Mycotoxins identification and quantitation analyses in grains were performed using AB Analyst software MultiQuant (version 3.0.2).

## Figures and Tables

**Table 1:** Calibration curve, linearity, LOD, LOQ, recovery and precision for 23 mycotoxins by grain (barley, wheat, oat).

Compound ^a^	Matrixs	Calibration Curve (μg/L)	R^2^	LOD (μg/kg)	LOQ (μg/kg)	Level 1 ^b^		Level 2 ^c^		Level3 ^d^		Precision RSD (%)	
						Recovery (%)	RSDs (%)	Recovery (%)	RSDs (%)	Recovery (%)	RSDs (%)	Intra-Day (*n* = 3)	Inter-Day (*n* = 9)
AFL B_1_	Barley	0.25–5	0.999	0.04	0.13	77.6	5.7	77.6	5.7	71.9	3.1	3.4	8.1
	Wheat		0.998	0.04	0.12	86.0	7.0	85.1	2.0	83.9	1.5	3.5	7.9
	Oat		0.996	0.04	0.15	92.0	2.2	103.5	10.1	94.3	3.8	5.4	8.3
AFL B_2_	Barley	0.25–5	1.000	0.06	0.20	78.0	9.2	79.5	3.5	72.3	5.4	6.1	8.4
	Wheat		1.000	0.05	0.18	79.3	9.5	84.3	1.5	83.1	2.7	4.5	7.1
	Oat		0.998	0.04	0.12	80.0	8.7	96.3	11.1	91.7	6.4	8.7	6.9
AFL G_1_	Barley	0.25–5	0.998	0.05	0.18	74.0	8.7	81.3	8.2	71.9	0.3	5.8	7.5
	Wheat		1.000	0.04	0.13	81.3	5.1	82.9	2.4	84.4	2.1	3.2	8.5
	Oat		0.997	0.04	0.12	86.7	5.3	106.1	10.6	94.0	6.4	7.4	5.1
AFL G_2_	Barley	0.25–5	0.999	0.04	0.14	85.3	4.9	81.9	5.0	71.5	6.6	5.5	11.7
	Wheat		0.999	0.04	0.12	78.7	5.9	89.6	2.7	90.5	4.2	4.2	9.8
	Oat		0.998	0.05	0.16	91.3	7.0	94.1	7.9	91.9	8.5	7.8	5.9
FB_1_	Barley	10–200	1.000	0.88	2.89	89.6	11.3	83.8	5.6	70.7	3.3	6.7	6.1
	Wheat		1.000	1.78	5.86	89.6	3.8	88.6	2.1	87.1	1.1	2.3	7.6
	Oat		0.997	1.15	3.81	86.9	7.6	97.6	9.8	92.1	8.0	8.4	6.5
FB_2_	Barley	10–200	0.999	1.92	6.34	79.6	7.5	83.8	5.1	70.7	4.8	5.8	4.5
	Wheat		1.000	1.36	4.48	93.4	1.7	90.2	2.8	85.6	1.1	1.9	7.0
	Oat		0.997	1.46	4.80	83.8	9.1	98.9	9.0	89.6	7.2	8.5	5.4
OTA	Barley	0.625–12.5	0.998	0.11	0.38	83.5	10.5	83.4	3.9	71.8	5.7	6.7	5.4
	Wheat		0.999	0.12	0.41	88.0	3.3	84.6	1.3	80.1	2.1	2.2	5.9
	Oat		0.996	0.10	0.32	90.4	6.7	100.9	7.9	92.7	3.0	5.9	7.4
DON	Barley	25–500	0.998	1.63	5.37	79.2	1.4	89.2	4.8	77.5	3.3	3.2	6.4
	Wheat		1.000	0.68	2.23	77.9	7.7	80.3	4.6	78.0	1.9	4.7	9.7
	Oat		0.997	2.17	7.15	73.0	5.7	89.2	5.5	83.9	6.1	5.8	7.6
HT-2	Barley	1.25–25	0.997	0.16	0.52	74.9	2.5	81.0	6.9	71.5	7.1	5.3	5.0
	Wheat		1.000	0.25	0.83	98.1	6.3	98.0	1.0	96.0	2.5	3.3	8.3
	Oat		0.997	0.09	0.31	86.8	11.4	92.6	8.9	86.8	7.9	9.4	6.8
T-2	Barley	1.25–25	0.998	0.24	0.79	76.4	7.4	81.4	9.1	72.2	5.7	7.4	9.4
	Wheat		1.000	0.24	0.78	71.1	3.2	71.7	2.2	70.4	1.7	1.6	8.0
	Oat		0.998	0.24	0.81	72.0	7.7	96.1	9.2	90.2	6.2	7.7	8.4
Em	Barley	0.5–10	0.998	0.12	0.38	73.7	7.5	87.9	6.2	87.9	4.0	5.9	9.3
	Wheat		1.000	0.08	0.27	75.7	3.3	74.5	0.3	73.9	4.7	2.8	5.8
	Oat		0.995	0.05	0.16	78.3	6.0	89.2	9.9	78.5	8.6	8.6	9.3
Emn	Barley	0.5–10	1.000	0.10	0.31	77.3	11.6	82.8	3.4	74.9	6.7	7.2	5.7
	Wheat		1.000	0.06	0.19	78.0	5.9	81.1	3.7	77.4	2.2	3.9	8.7
	Oat		0.994	0.07	0.22	70.3	7.2	92.1	11.5	83.2	6.3	8.3	7.4
Es	Barley	0.5–10	0.995	0.07	0.24	104.0	7.3	93.3	1.1	87.7	4.4	4.2	6.1
	Wheat		0.999	0.07	0.24	98.7	7.7	92.3	3.9	101.5	1.2	4.3	9.0
	Oat		0.998	0.11	0.36	76.0	11.4	91.5	10.9	81.0	5.3	9.2	8.4
Esn	Barley	0.5–10	0.999	0.06	0.20	88.3	11.8	93.3	1.1	70.3	6.6	6.5	10.7
	Wheat		0.996	0.08	0.27	86.3	6.6	92.3	3.9	86.4	2.1	4.2	8.3
	Oat		0.997	0.10	0.32	75.7	7.3	91.5	10.9	90.5	6.7	8.3	8.7
Et	Barley	0.5–10	1.000	0.11	0.36	81.3	8.6	86.9	11.9	73.4	2.7	7.8	7.0
	Wheat		0.999	0.07	0.24	87.0	4.1	83.7	6.5	75.5	3.5	4.7	8.2
	Oat		0.995	0.10	0.32	95.7	12.1	107.7	11.5	91.9	9.9	11.2	7.4
Etn	Barley	0.5–10	1.000	0.11	0.37	88.3	6.8	85.5	10.5	73.8	7.0	8.1	8.6
	Wheat		0.999	0.10	0.33	90.7	5.0	85.6	3.4	84.3	1.7	3.4	7.6
	Oat		0.997	0.10	0.31	83.3	5.4	106.1	12.5	91.3	4.5	7.5	9.1
Eco	Barley	0.5–10	0.999	0.03	0.10	91.3	10.8	83.9	1.5	73.6	7.2	6.5	8.8
	Wheat		1.000	0.08	0.25	76.0	3.5	90.1	7.5	81.9	1.6	4.2	8.5
	Oat		0.996	0.11	0.35	88.7	4.0	104.1	8.5	92.0	4.3	5.6	10.4
Econ	Barley	0.5–10	0.998	0.11	0.37	81.0	8.6	81.2	6.3	73.3	8.3	7.7	6.2
	Wheat		0.995	0.10	0.34	83.7	2.5	88.1	8.5	86.4	5.5	5.5	10.0
	Oat		0.995	0.08	0.27	70.7	2.9	97.1	9.9	97.5	7.5	6.8	6.5
Ecy	Barley	0.5–10	0.999	0.11	0.38	83.7	11.1	81.2	7.7	70.1	6.9	8.7	8.2
	Wheat		1.000	0.10	0.35	88.3	6.2	86.9	3.4	82.6	3.0	4.2	9.3
	Oat		0.996	0.09	0.29	80.3	10.9	99.7	9.9	96.9	4.0	8.3	8.0
Ecyn	Barley	0.5–10	1.000	0.12	0.39	88.7	11.7	81.7	6.1	71.1	5.8	7.9	7.6
	Wheat		1.000	0.06	0.20	86.0	10.1	88.0	0.9	84.5	1.8	4.3	8.2
	Oat		0.996	0.10	0.32	82.0	4.9	109.3	4.2	95.1	3.9	4.3	7.0
Ecr	Barley	0.5–10	0.999	0.04	0.12	97.0	6.8	82.9	7.7	72.3	5.2	6.6	6.5
	Wheat		1.000	0.07	0.24	84.3	3.8	84.3	3.2	81.6	1.5	2.8	8.0
	Oat		0.999	0.09	0.31	78.0	4.6	98.9	6.9	90.3	6.4	6.0	6.9
Ecrn	Barley	0.5–10	1.000	0.09	0.31	88.3	7.5	82.3	11.4	70.7	7.9	9.0	7.4
	Wheat		1.000	0.11	0.36	87.0	3.0	84.5	1.9	82.8	3.2	2.7	7.2
	Oat		0.998	0.07	0.25	82.3	9.1	100.9	11.4	93.2	4.4	8.3	8.4
ZEN	Barley	2.5–50	0.999	0.15	0.49	71.4	6.4	79.0	3.6	71.0	3.1	4.1	6.0
	Wheat		0.991	0.25	0.82	104.7	4.7	83.1	2.3	73.9	3.2	3.4	6.2
	Oat		0.997	0.40	1.32	90.4	7.1	107.2	8.8	98.6	7.1	7.7	6.0

^a^ AFL B_1_: aflatoxin B_1_, AFL B_2_: aflatoxin B_2_, AFL G_1_: aflatoxin G_1_, AFL G_2_: aflatoxin G_2,_ FB_1_: fumonisin B_1_, FB_2_: fumonisin B_2_, OTA: ochratoxins A, DON: deoxynivalenol, ZEN: zearalenone, HT-2: HT-2 toxin, T-2: T-2 toxin, Em: ergometrine, Emn: ergometrinine, Es: ergosine, Esn: ergosinine, Et: ergotamine, Etn: ergotaminine, Eco: ergocornine, Econ: ergocorninine, Ecy: ergocryptine, Ecyn: ergocryptinine, Ecr: ergocristine, Ecrn: ergocristinine. ^b^ 2 μg/kg for AFL B_1_, AFL B_2_, AFL G_1_, AFL G_2_; 80 μg/kg for FB_1_, FB_2_; 5 μg/kg for OTA; 200 μg/kg for DON; 20 μg/kg for ZEN; 10 μg/kg for HT-2, T-2; 4 μg/kg for EAs; ^c^ 2.5 times of level 1; ^d^ 5 times of level 1.

**Table 2:** Incidence, concentration range and mean of 23 mycotoxins in positive samples (μg/kg).

Compound ^a^	Barley (*n* = 95)		Wheat (*n* = 19)		Oat (*n* = 29)		Total (*n* = 143)	
	Incidence (%)	Concentration Range (µg/kg)	Incidence (%)	Concentration Range (µg/kg)	Incidence (%)	Concentration Range (µg/kg)	Incidence (%)	Concentration Range (µg/kg)
AFL B_1_	ND ^b^	-	ND ^b^	-	ND ^b^	-	ND^b^	-
AFL B_2_	ND	-	ND	-	ND	-	ND	-
AFL G_1_	ND	-	ND	-	ND	-	ND	-
AFL G_2_	ND	-	ND	-	ND	-	ND	-
FB_1_	ND	-	ND	-	ND	-	ND	-
FB_2_	ND	-	ND	-	ND	-	ND	-
OTA	ND	-	ND	-	ND	-	ND	-
DON	21.05	11.28–112.72	31.58	5.96–37.33	ND	-	18.18	<LOQ-112.72
HT-2	15.79	0.56–0.68	ND	-	3.45	<LOQ-40.64	11.19	<LOQ-40.64
T-2	ND	-	ND	-	3.4	<LOQ-13.84	0.7	<LOQ-13.84
Em	ND	-	ND	-	ND	-	ND	-
Emn	ND	-	ND	-	ND	-	ND	-
Es	1.05	<LOQ-0.72	ND	-	ND	-	0.70	<LOQ-0.72
Esn	ND	-	ND	-	ND	-	ND	-
Et	ND	-	5.3	<LOQ	ND	-	0.7	<LOQ
Etn	ND	-	ND	-	ND	-	ND	-
Eco	1.05	<LOQ-0.16	ND	-	ND	-	0.70	<LOQ
Econ	ND	-	ND	-	ND	-	ND	-
Ecy	ND	-	ND	-	ND	-	ND	-
Ecyn	ND	-	ND	-	ND	-	ND	-
Ecr	1.05	<LOQ-0.72	ND	-	ND	-	0.70	<LOQ-0.72
Ecrn	ND	-	ND	-	ND	-	ND	-
ZEN	8.42	2.64–12.40	10.53	5.68–10	ND	-	6.99	2.64–12.40

^a^ AFL B_1_: aflatoxin B_1_, AFL B_2_: aflatoxin B_2_, AFL G_1_: aflatoxin G_1_, AFL G_2_: aflatoxin G_2,_ FB_1_: fumonisin B_1_, FB_2_: fumonisin B_2_, OTA: ochratoxins A, DON:deoxynivalenol, ZEN: zearalenone, HT-2: HT-2 toxin, T-2: T-2 toxin, Em: ergometrine, Emn: ergometrinine, Es: ergosine, Esn: ergosinine, Et: ergotamine, Etn: ergotaminine, Eco: ergocornine, Econ: ergocorninine, Ecy: ergocryptine, Ecyn: ergocryptinine, Ecr: ergocristine, Ecrn: ergocristinine. ^b^ ND: not detectable.

## Data Availability

Not applicable.

## Supplementary Materials

The following supporting information can be downloaded at: https://www.mdpi.com/article/10.3390/toxins14070457/s1, Figure S1: Chemical structures of the 23 target mycotoxins; Figure S2: Qualitative and quantitative ion chromatograms of 23 mycotoxin analytes spiked in blank wheat samples used in this study using HPLC-MS/MS (2 μg/kg for AFB_1_, AFB_2_, AFG_1_, AFG_2_; 80 μg/kg for FB_1_, FB_2_; 5 μg/kg for OTA; 200 μg/kg for DON; 20 μg/kg for ZEN; 10 μg/kg for HT-2, T-2; 4 μg/kg for EAs); Table S1: Compounds analyzed in grains (barley, oat and wheat) with the legal status and mass spectrometric conditions; Table S2: Calibration ranges, concentration of the recovery test and lowest limit of grains (barley, oat and wheat); Table S3: Concentrations of 23 mycotoxins in positive samples (μg/kg).
