# Supplementary material for: In-House Validation of an Efficient and Rapid Procedure for the Simultaneous Determination and Monitoring of 23 Mycotoxins in Grains in Korea
Source: Toxins (Basel). 2022 Jul 2;14(7):457. doi: 10.3390/toxins14070457 (PMC9318384; doi:10.3390/toxins14070457)
Supplement: Supplementary file 1 [file toxins-14-00457-s001.zip › toxins-1785393 supplementary material.pdf]

toxins

Article

In-house Validation of an Efficient and Rapid Procedure for the Simultaneous Determination and Monitoring of 23 Mycotoxins in Grains in Korea

Hyoyoung Kim, Eun Joo Baek, Byeung Gon Shin, Ho Jin Kim and Jang-Eok Kim

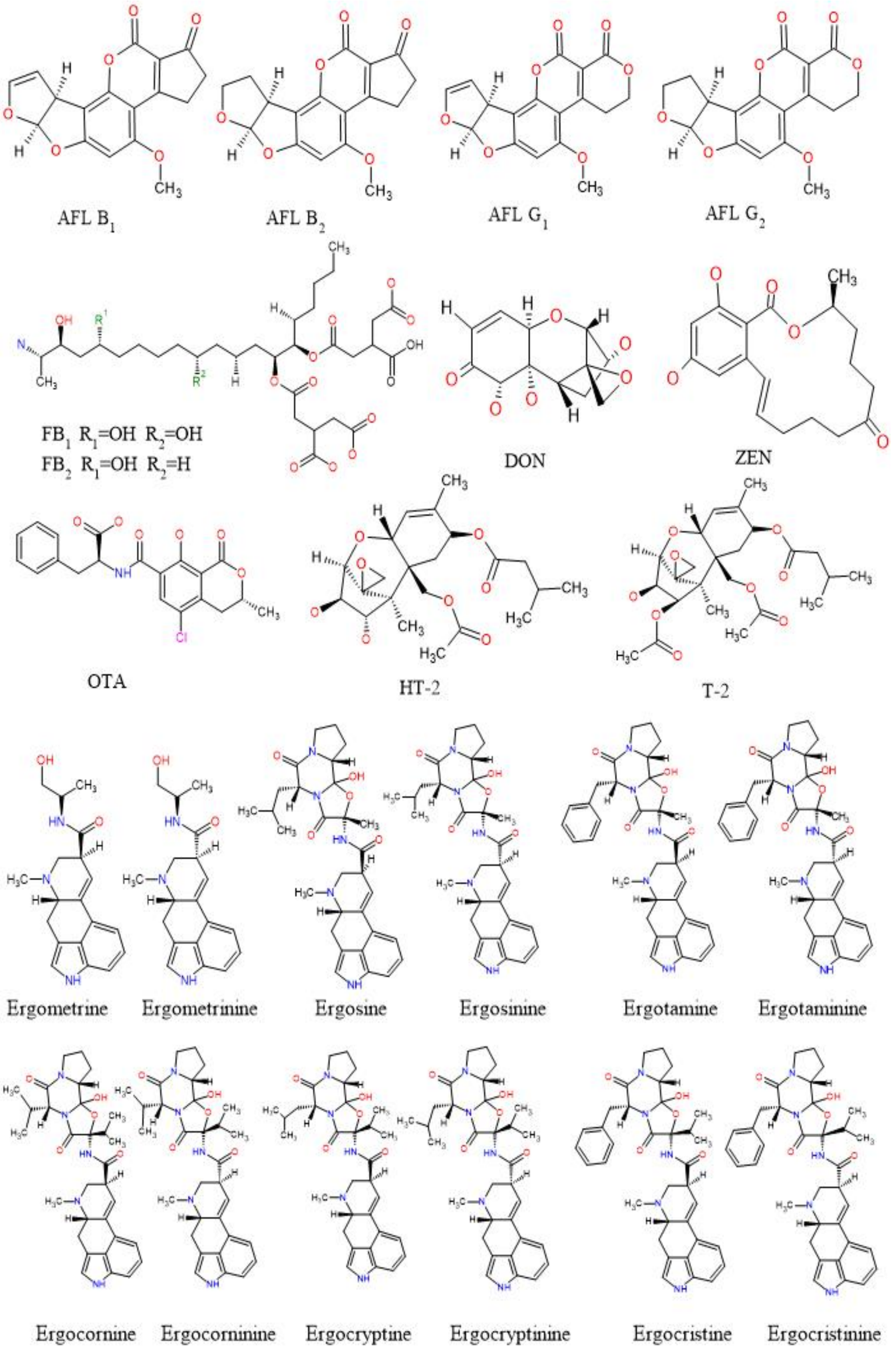

Figure S1. Chemical structures of the 23 target mycotoxins.

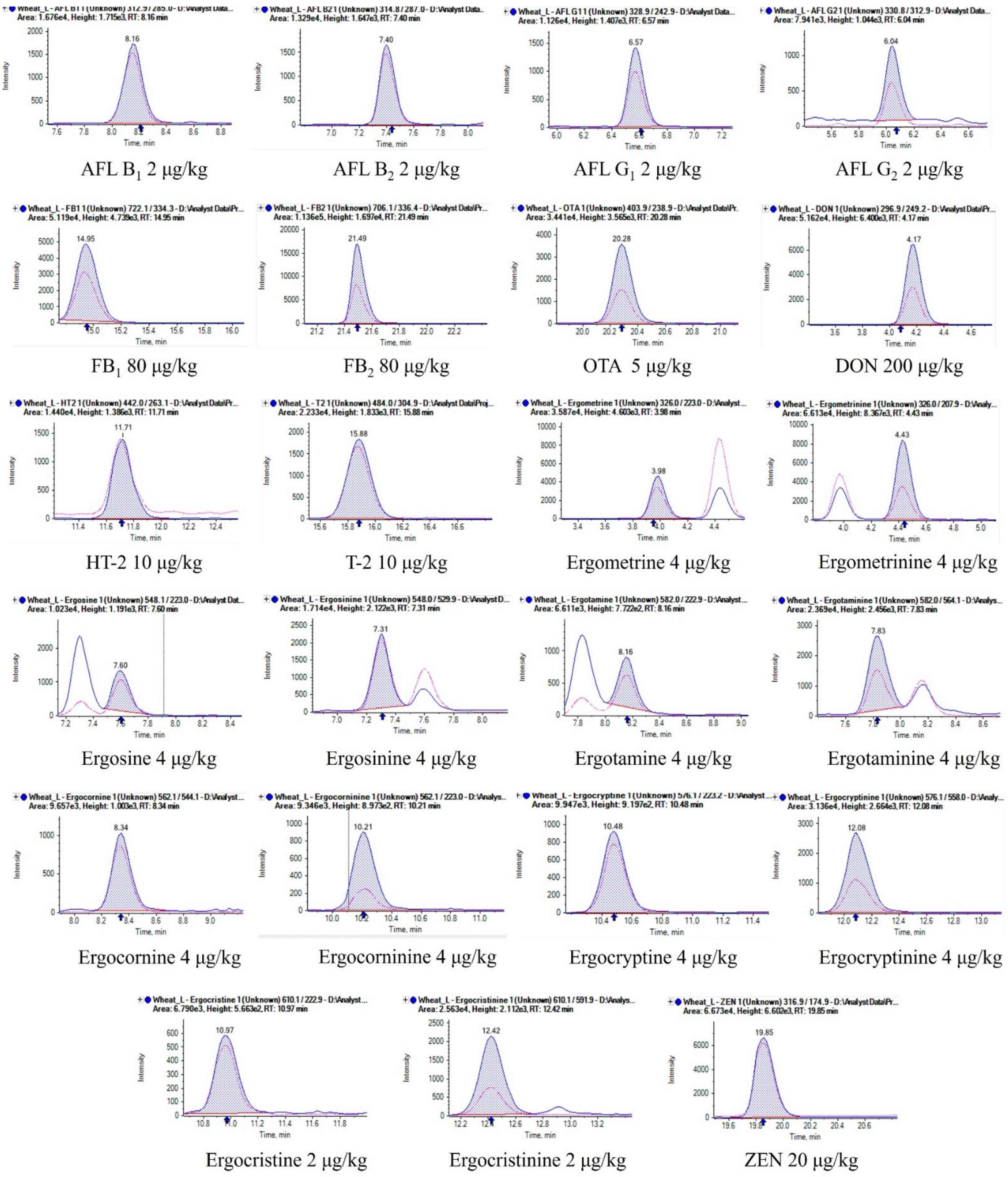

Figure S2. Qualitative and quantitative ion chromatograms of 23 mycotoxin analytes spiked in blank wheat samples used in this study using HPLC–MS/MS (2 μg/kg for AFB1, AFB2, AFG1, AFG2; 80 μg/kg for FB1, FB2; 5 μg/kg for OTA; 200 μg/kg for DON; 20 μg/kg for ZEN; 10 μg/kg for HT-2, T-2; 4 μg/kg for EAs).
